# Insulin icodec: A novel once-weekly formulation for the treatment of type 1 and type 2 diabetes mellitus

**DOI:** 10.1007/s11154-025-09960-x

**Published:** 2025-03-29

**Authors:** David Q. Pham, John Andraos, Joelle Ayoub

**Affiliations:** 1 Department of Pharmacy, College of Pharmacy, Western University of Health Sciences, Pomona, CA 91766 USA; 2 Department of Pharmacy, Hoag Hospital, Mary & Dick Allen Diabetes Center, Newport Beach, CA 92663 USA; 3 Department of Pharmacy, Cedars Sinai Medical Network, Beverly Hills, CA 90211 USA; 4 Department of Pharmacy, Pomona Valley Health Center, Pomona, CA 91767 USA

**Keywords:** Icodec, Insulin, Type 1 Diabetes, Type 2 Diabetes

## Abstract

Insulin icodec is a novel once-weekly basal insulin analog subcutaneous injection seeking approval by the United States Food and Drug Administration (FDA) for use in both type 1 and type 2 diabetes mellitus. The mission of this manuscript is to provide a thorough overview of insulin icodec’s clinical trials that were involved in its approval as well as review its pharmacology, pharmacokinetics, adverse effects, drug interactions, dosage recommendations, and regulatory issues. This article includes a thorough review of insulin icodec’s safety and efficacy in type 1 and type 2 diabetes mellitus including its pharmacokinetic and pharmacodynamic profile. A systematic search of the electronic database of PubMed from inception until December 2024 using MeSH keywords was completed. Keywords used were icodec, insulin, type 1 diabetes, and type 2 diabetes. Overall, 14 clinical trials were identified and reviewed. The majority of the trials reviewed showed decreases in A1C as primary endpoints and non-inferiority and superiority with insulin icodec versus the comparator. In select studies, mild hypoglycemia was more evident in subjects taking insulin icodec versus the comparator but no other concerns were identified. The reviewed literature showed similar and sometimes improved glycemic control when insulin icodec was compared to other long-acting insulins both in insulin-naive and previously insulin-treated patients. Hypoglycemia was similar or slightly increased with insulin icodec when compared to other long acting insulins. Overall, icodec is a useful, new formulation of basal insulin that allows for less injections, improved compliance, and potentially improved glycemic control providing a new tool to practitioners managing patients with diabetes who need to be on insulin.

## Introduction

### Background

Insulin recently celebrated 100 years since its discovery in 1921, yet limitations of insulin therapy still remain. The first limitations is the need to administer insulin as an injectable, continued risk of hypoglycemia, and the high frequency of its administration. While the risk of hypoglycemia has been improved through the use of insulin analogs, the frequency of insulin injections has remained at once daily since the approval of insulin glargine 24 years ago [1]. Since the inception of basal insulin, many attempts have been made to increase the half-life to reduce daily dosages. The initial formulas needed to be injected several times a day and our current longest acting basal insulin needs to be injected once daily. In diabetes management, once-weekly injectables are favored over once-daily injectables in areas of glycemic control, improved quality of life, compliance, and patient satisfaction [2]. Insulin icodec is a novel ultra-long acting basal insulin that is administered once-weekly which may improve convenience, adherence, and quality of life [3]. It is currently seeking approval in the United States Food and Drug Administration (FDA) for use in both type 1 diabetes (T1DM) and type 2 diabetes mellitus (T2DM). Since icodec is the world’s first once-weekly insulin, a thorough review of its safety and efficacy is warranted. The mission of this manuscript is to provide a thorough overview of insulin icodec’s clinical trials that were involved in its approval as well as review its pharmacology, pharmacokinetics, adverse effects, drug interactions, dosage recommendations, and regulatory issues.

### Pharmacology

T1DM occurs due to the dysfunction of pancreatic β-cells, causing a shortage of systemic insulin, while T2DM is defined as insulin resistance, with the cells desensitized from signal transduction and mitochondrial exhaustion along with a gradual decline in pancreatic B-cell function. Deficits of insulin secretion and insulin intolerance play cardinal roles in the pathogenesis of T1DM and T2DM. To treat these conditions, insulin sources, formulations and duration have evolved tremendously over the last century. Insulin is released by the pancreas in response to blood glucose and binds to insulin receptors on cell membranes allowing glucose to enter the cell to be used as a source of energy. To emulate the healthy physiology in the human body, insulin analogs are available as prandial insulins and basal insulins. Prandial insulin analogs compensate for the spike in blood glucose where high insulin demand is needed after meals with progressive diabetes. Basal insulin analogs are long-acting insulins and work to provide constant insulin levels and stabilize the blood glucose level between meals and overnight [4]. In the body, endogenous insulin is released slowly, similar to the mechanism of an insulin pump that secretes rapid-acting insulin constantly throughout the day. However, exogenous basal insulin was produced to prolong insulin action through varying amounts of zinc and protamine which slowed absorption and reduced solubility after subcutaneous injection.

Endogenous insulin has a biologic half-life of a few minutes before it is quickly degraded [5]. However, pharmacologic insulins alter the distribution phase of insulin allowing for lengthened durations of activity. Previous long-acting insulins that are taken less frequently utilize binding or alterations of molecules to extend the presence of insulin in the body. Neutral Protamine Hagedorn (NPH) insulin is created externally by cocrystallization of molecules of insulin with zinc to create a complex that allows for slower release of the insulin once injected in the body [6]. In 2000, insulin glargine was created by adding two arginine residues to the carboxy-terminal of the B chain increasing positive charge of the molecule leading to decreased solubility so that insulin would micro-precipitate after injection increasing its half-life [7]. Insulin detemir was created in 2005, in addition of a C-14 fatty acylated acid to the insulin which also slowed absorption and formed non-covalent lipidated reversible insulin albumin complex with human albumin [8]. This provided a lower glycemic variability compared to insulin glargine, however diabetes patients sometimes can still require twice daily doses of daily basal insulin for glycemic control. Insulin degludec was then created with another addition of a C-16 fatty acid, along with zinc and phenolic preservative for a longer duration of action of 42 h in 2015 [9]. In the same year, insulin glargine 300 units per ml was formulated to slow dissolution of micro-precipitates prolonging insulin glargine action to > 30 h. Most recently in the last few years, insolin icodec is pending approval as the first weekly insulin with a prolonged duration of action. This modification allows for slow release of insulin over eight days, which enables its once-weekly dosing.

### Pharmacokinetics

Insulin icodec is a new once-weekly insulin seeking FDA approval. Similar to insulin glargine, insulin icodec utilizes alterations of amino acids to create depots for insulin in the body. Icodec contains three substitutions to the amino acid structure and attaches miniPEG-gGlu linked-1, 20-icosanedioic acid (C20) at position LysB29. This fatty diacid chain allows for binding of insulin to albumin resulting in an inactive depot that circulates and is slowly released allowing for a longer duration of action. Insulin icodec also has a critical substitution (B16 Tyr/His) which causes a significant reduction in insulin receptor affinity, allowing for reduced receptor binding that minimizes insulin receptor-mediated clearance and that leads to a further increase in the duration of action [1, 10].

These alterations allow for an insulin with a half-life of 196 h and a very stable release allowing patients to take it once a week. Peak serum concentration levels occurred at 16 h and were not remarkably higher than serum levels at other times after injection. Distribution occurred fairly evenly throughout the week ranging from 12% of the weekly dose (day 7) to 16% of the weekly dose (day 3). Of note, patients did not achieve steady state until 3–4 injections which is an important clinical consideration and is likely why a loading dose of insulin icodec is recommended [11]. These pharmacokinetic characteristics (distribution of insulin release, peak insulin concentrations) are translated into clinical outcomes (glucose lowering effects, hypoglycemic events) in several studies that are summarized below.

## Methods

A systematic search of the electronic database of PubMed from inception until December 2024 using MeSH keywords was completed. Ongoing trials of insulin icodec in diabetes were also searched with keywords used were “icodec AND type 1 diabetes” and “icodec AND type 2 diabetes”. The 48 articles of the search were filtered by “randomized controlled trials” resulting in 17 trials that were identified. Of the 17, three articles were commentaries of studies and therefore excluded resulting in 14 studies reviewed. The most notable trials included the ONWARDS clinical program [12], which were a group of trials with real-world elements that involved more than 4,000 adults with type 1 or type 2 diabetes.

## Results & discussion

### Clinical trial reviews

**Fig. 1:**
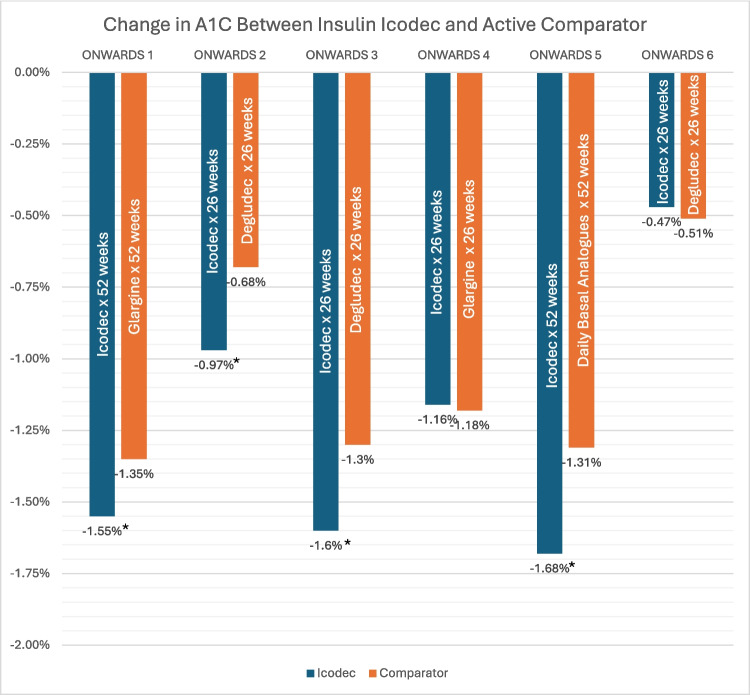
Change in A1C Between Insulin Icodec and Active Comparator [16–21]

Fourteen clinical trials evaluated the pharmacokinetic profile, efficacy, as well as safety of insulin icodec. Various results included pharmacokinetics, dosing, and outcomes such as change in A1C, continuous glucose monitoring (CGM) parameters, weight gain, and hypoglycemic episodes. Details and results of the studies are shown in Table 1, Fig. 1, and discussion of the trials are summarized below.

**Table 1:** Study results

Publication	Design	Aim	Results Summary
Nishimura et al. [11]	*N* = 38	Primary Purpose: Describe the molecular engineering, biological, and pharmacological properties of insulin icodec (half-life and glucose-lowering effect)	Mean half-life: 196 h Median time to Cmax: 16 h AUC 0–168 h vs Dose: 1 1 (95% CI, 0.56 to 1.10)
Arms: Insulin icodec 12 nmol/kg (*n* = 13), 20 nmol/kg (*n* = 13) or 24 nmol/kg (*n* = 12)	Randomized, double-blind, double-dummy, active-controlled, multiple-dose, dose escalation trial		
Population: Type 2 DM			
Published: 2021	Duration: N/A		
Pieber et al. [13]	*N* = 46	Primary Endpoint:* In vivo* pharmacokinetic profile of insulin icodec in humans with T2DM	Time to steady state (pharmacokinetic modeling): 3–4 doses/weeks Daily proportions of glucose lowering effects at steady state: Day 1: 14.1% Day 2: 16.1% Day 3: 15.8% Day 4: 15.0% Day 5: 14.0% Day 6: 13.0% Day 7: 12.0%
Arms:	Open-label pharmacokinetic/pharmacodynamic trial		
Population: T2DM			
Published: 2023	Duration:		
Plum-Mörschel et al. [10]	*N* = 25	Primary Endpoint: Insulin blood levels and glucose lowering effects based on location of subcutaneous insulin icodec injection	Total insulin exposure (AUC): Abdomen/thigh: 1.02 (95% CI, 0.96 to 1.09) Upper arm/thigh: 1.04 (95% CI, 0.98 to 1.10) Abdomen/upper arm: 0.98 (95% CI, 0.93 to 1.05) Maximum concentration (Cmax): Abdomen/thigh: 1.17 (95% CI, 1.07 to 1.29, *p* < 0.05) Upper Arm/thigh: 1.24 (95% CI, 1.14 to 1.35, *p* < 0.05) Abdomen/upper arm: 0.94 (95% CI, 0.85 to 1.03) Glucose-lowering effect (AUC glucose infusion rate from 36–60 h during glucose clamp): Thigh: 1961 mg/kg Abdomen: 2130 mg/kg Upper Arm: 2391 mg/kg
Arms: SC injection (abdomen), SC injection (thigh), SC injection (arm)	Randomized, open-label, crossover pharmacokinetic/pharmacodynamic trial		
Population: T2DM			
Published: 2023	Duration: N/A		
Bajaj et al. [14]	*N* = 154	Primary end point: percentage of TIR (3.9–10.0 mmol/L [70–180 mg/dL]) during the last 2 weeks of treatment (weeks 15 and 16), measured by CGM Supportive secondary end points: Changes from baseline to week 16 in HbA1c, fasting plasma glucose (FPG), and body weight; weekly insulin dose during the last 2 weeks of treatment (weeks 15 and 16); and number of on-treatment adverse events (AEs) from baseline to week 21 and number of self-reported hypoglycemic episodes documented by SMBG or assessed as the requirement of external assistance for recovery	TIR at week 15 and 16: Icodec LD: 72.9% Icodec non-loading dose (NLD): 66.0% IGlar U100: 65.0% Changes from baseline in TIR of Icodec LD: 15.4%-points (3 h 42 min/day) Icodec NLD: 8.6%-points, (2 h 4 min/day) IGlar 100: 7.6%-points, (1 h 49 min/day) Icodec LD group compared with the IGlar U100 group (estimated treatment difference [ETD], 7.88%-points [95% CI 1.83 to 13.93]; *P* = 0.01) Mean A1c change at week 16: Icodec LD: −0.8% (−8.4 mmol/mol) Icodec NLD: −0.5% (−5.2 mmol/mol), IGlar U100: −0.5% (−5.9 mmol/mol) No statistically significant differences between icodec groups and IGlarU100 FPG at week 16: Icodec LD: −0.7 mmol/L Icodec NLD: −0.8 mmol/L Iglar U1000: −0.6 mmol/L Groups were not statistically significantly different from each other. Further details can be found in the study
Arms: Those receiving once-daily basal insulin (except for those receiving IGlar U300) at baseline underwent a “unit to unit” switch to icodec (700 units/mL; prefilled pen injector) or IGlar U100 (100 units/mL) based on their daily dose. For those who had been receiving twice-daily basal insulin or once-daily IGlar U300 at baseline, Participants in the icodec loading dose (LD) group received an initial 100% loading dose with the first dose of icodec (i.e., the first weekly dose was doubled), and then it was reverted to the calculated weekly dose at week 2	multicenter, open-label, treat-to-target phase 2 trial randomized, Phase 2 trial		
Population: T2DM, Inadequately Controlled on Basal Insulin - (HbA1c 7.0–10.0% [53.0–85.8 mmol/mol])			
Published: 2021	Duration: 16-week, treat-to-target study. Insulin-naive adults (*n* = 205) with T2DM and HbA1c 7–10% while treated with oral glucose-lowering medications initiated once-weekly icodec titrations		
Lingvay et al. [15]	*N* = N/A	Primary end point: percentage of TIR (3.9–10.0 mmol/L [70–180 mg/dL]) during the last 2 weeks of treatment (weeks 15 and 16), measured by CGM Supportive secondary end points: Changes from baseline to week 16 in HbA1c, fasting plasma glucose (FPG), and body weight; weekly insulin dose during the last 2 weeks of treatment (weeks 15 and 16); and number of on-treatment adverse events (AEs) from baseline to week 21 and number of self-reported hypoglycemic episodes documented by SMBG or assessed as the requirement of external assistance for recovery	TIR improved from baseline to weeks 15 and 16: Icodec A: 57% → 76.6% Icodec B: 55.2% → 83.0% Icodec C: 51% → 80.9% IGlar U100: 55.3% → 75.9% TIR was longer for titration B than for IGlar U100 (estimated treatment difference (ETD) 7.08%-points; 95% CI 2.12 to 12.04; *P* = 0.005) TIR was greater for icodec titration C Glar U100, (ETD 5.01%-points; 95% CI –0.04 to 10.05; *P* = 0.05) Estimated mean changes from baseline to week 16 in HbA1c: Icodec titrations A: –1.0%-point (–10.9 mmol/mol), Icodec titration B: –1.2%-points (–13.4 mmol/mol), Icodec titrationC: –1.4%-points (–15.1 mmol/mol), IGlar U100: –1.0%-point (–11.1 mmol/mol) Other secondary endpoint details can be found in the Supplementary index of the study
Arms: Patients were randomized 1:1:1:1 to receive subcutaneous injections of once-weekly insulin icodec following one of three titration algorithms (icodec titrations A, B, or C, outlined below) or once-daily IGlar U100 icodec titrations A (prebreakfast self-measured blood glucose target 80–130 mg/dL; adjustment ± 21 units/week; *n* = 51), B (80–130 mg/dL; ± 28 units/week; *n* = 51), or C (70–108 mg/dL; ± 28 units/week; *n* = 52), or once-daily insulin glargine 100 units/mL (IGlar U100) (80–130 mg/dL; ± 4 units/day; *n* = 51)	randomized, active-controlled, parallel-group, multicenter, multi-national, open-label, phase 2, treat-to-target trial conducted in seven countries		
Population: adults aged 18–75 years who received a diagnosis of T2DM at least 180 days prior to screening, were treated with metformin with or without dipeptidyl peptidase 4 inhibitor (DPP4i) and/or sodium–glucose cotransporter 2 inhibitors (SGLT2i), and had glycated hemoglobin (HbA1c) of 7.0–10.0% (53.0– 85.8 mmol/mol)			
Published: 2021	Duration: 16-week, treat-to-target study. Insulin-naive adults (*n* = 205) with T2DM and HbA1c 7–10% while treated with oral glucose-lowering medications initiated once-weekly icodec titrations		
Philis-Tsimikas et al. [12] Rationale and Design of ONWARDS trials	*N* = N/A	Primary purpose: Describe the rationale of the series of phase 3a trials (ONWARD 1–6) of insulin icodec	Major outcomes: Glycemic (change in A1C from baseline to week 26 or week 52, FBG, time in glycemic range) and safety outcomes (hypoglycemia): All studies Digital dose titration: ONWARD-5 Population: Insulin-naive: ONWARDS 1, 3, 5 Insulin-treated: ONWARDS 2, 4 T1DM: ONWARDS 6 Comparator: Glargine U-100: ONWARDS 1, 4, 5 Glargine U-300: ONWARDS 5 Degludec: ONWARDS 2, 3, 5, 6
Arms:	Design paper		
Population:			
Published: 2023	Duration: N/A		
Rosenstock et al. [16] ONWARDS 1	*N* = 984	Primary endpoint: Change in A1C baseline to week 52 Secondary endpoint: Percent TIR in weeks 48–52. Hypoglycemic events recorded baseline to weeks 52 and 83	A1C reduction at 52 weeks: Icodec: −1.55% Glargine: −1.35% (95% CI, −0.36 to −0.03) Noninferiority [margin + 0.3]: (*P* < 0.001) Superiority: (*P* = 0.02) TIR: Icodec: 71.9% Glargine: 66.9% (95% CI, 1.92 to 6.62, *P* < 0.001) Clinically significant/severe hypoglycemia at 52 weeks: Icodec: 0.30 events per person-year Glargine: 0.16 per person-year (95% CI, 0.98 to 2.75) Clinically significant/severe hypoglycemia at 83 weeks: Icodec: 0.30 events per person-year Glargine: 0.16 per person-year (95% CI, 1.02 to 2.61)
Arms: Icodec vs glargine U100	Randomized, open-label, multicenter, treat-to-target, phase 3 trial		
Population: T2DM, insulin-naive			
Published: 2023	Duration: 78 weeks (52 main, 26 extension), 5 week follow up period		
Philis-Tsimikas et al. [17] ONWARDS 2	*N* = 526	Primary endpoint: change in A1C baseline to week 26 Secondary endpoint: CGM-based double blinded, 36 point DTSQ score, and adverse events	A1C reduction at 26 weeks: Icodec: −0.97% Degludec: −0.68% (95% CI, −0.37 to −0.08) Noninferiority [margin + 0.3]: (*P* < 0.0001) Superiority: (*P* = 0.0028) Weight at 26 weeks: Icodec: + 1.40 kg Degludec: −0.30 kg (95% CI, −0.76 to 2.63). Rates of combined levels 2 & 3 hypoglycemia were less than one event per patient-year for both groups (95% CI, 0.93 to 4.02) Treatment Satisfaction DTSQ: Icodec: 4.22 Degludec: 2.96 (95% CI, 0.41 to 2.10) Adverse Event: Icodec: 61% Degludec: 51%
Arms: Icodec vs. daily degludec	Randomized, open-label, multicenter (9 countries, 71 locations), treat-to-target		
Population: Type 2 subjects, previously on basal insulin with or without other antidiabetic agents			
Published: 2023	Duration: 26 weeks		
Lingvay et al. [18] ONWARDS 3	*N* = 588	Primary endpoint: Change in A1C baseline to week 26 Secondary endpoint: Change in FPG baseline to week 26, weight change baseline to week 26, mean insulin dose in last 2 weeks of study, and number of level 2 and 3 hypoglycemic episodes	A1C reduction at 26 weeks: Icodec: −1.6% Degludec: −1.3% (95% CI, −0.3 to −0.1) Noninferiority [margin + 0.3]: (*P* < 0.001) Superiority: (*P* = 0.002) FPG at 26 weeks: Icodec: −54 mg/dl Degludec: −54 mg/dl (95% CI, −6 to 5) Weight at 26 weeks: Icodec: + 2.8 kg Degludec: + 2.3 kg (95% CI, −0.19 to 1.10) Mean insulin dose in last 2 weeks of study: Icodec: 204 u/week or 29 u/d Degludec: 187 u/week or 27 u/d (95% CI, 0.98 to 1.22) Combined level 2 or 3 hypoglycemia rates at 26 weeks: Icodec: 0.31 events per patient-year Degludec: 0.15 events per patient-year (95% CI, 0.87 to 3.80)
Arms: Icodec vs. daily delgudec	Randomized, double blind, treat-to-target		
Population: Insulin naive type 2 already on non-insulin antidiabetic treatment			
Published: 2023	Duration: 26 weeks		
Mathieu et al. [19] ONWARDS 4	*N* = 582	Primary endpoint: Change in A1C baseline to week 26 Secondary endpoints: Change in FPG baseline to 26 weeks, percent TIR 70–180 mg/dL from week 22 to 26, mean weekly insulin dose from week 24 to 26, change in weight baseline to week 26, level 2 and 3 hypoglycemia	A1C reduction at 26 weeks: Icodec: −1.16% Glargine: −1.18% (95% CI, −0.11 to 0.15) Noninferiority [margin + 0.3]: (*P* < 0.0001) FPG at 26 weeks: Icodec: −32 mg/dl Glargine: −29 mg/dl (95% CI, −10.59 to 5.63) TIR: Icodec: 66.9% Glargine: 66.4% (95% CI, −2.52 to 3.09, *P* = 0.84) Mean insulin dose in last 2 weeks of study: Icodec: 514 u/week or 73 u/d Glargine: 559 u/week or 80 u/d (95% CI, 0.85 to 0.99) Change in weight at 26 weeks: Icodec: + 2.7 kg Glargine: + 2.2 kg (95% CI, −0.39 to 1.54) Combined level 2 or 3 hypoglycemia rates at 26 weeks: Icodec: 0.51 events per patient-year Degludec: 0.56 events per patient-year (95% CI, 0.73 to 1.33)
Arms: Switching to icodec vs. daily glargine with mealtime aspart (2–4 times daily)	Randomized, open-label, multicenter, non-inferiority, treat-to-target		
Population: Type 2 previously on basal-bolus therapy			
Published: 2023	Duration: 26 weeks		
Bajaj et al. [20] ONWARDS 5	*N* = 1,085	Primary endpoint: A1C change from baseline to week 52 Secondary endpoints: TRIM-D* compliance domain score and change DTSQ** satisfaction score	A1C reduction at at 52 weeks: Icodec with app: −1.68% Daily basal analogues: −1.31% (95% CI, −1.55 to −1.07) Noninferiority [margin + 0.3]: (*P* < 0.001) Superiority: (*P* = 0.009) TRIM-D* compliance domain score at week 52: Icodec with app: 90.42 Daily basal analogues: 87.37 (95% CI, 1.28 to 4.81) DTSQ** total treatment satisfaction score at week 52: Icodec with app: 4.68 Daily basal analogues: 3.90 (95% CI, 0.10 to 1.47)
Arms: Icodec with dosing guide app vs. daily basal insulin analogues	Randomized, multi-center, open-label, parallel-group		
Population: Insulin-naive subjects with T2DM			
Published: 2023	Duration: 52 weeks		
Russell-Jones et al. [21] ONWARDS 6	*N* = 582	Primary endpoint: change in HbA1c from baseline to week 26 Secondary efficacy endpoints: change in HbA1c from baseline to week 52 (to include the 26-week extension phase) change in fasting plasma glucose (FPG) from baseline to week 26 percentage of TIR; 3·9–10·0 mmol/L [70–180 mg/dL]) during weeks 22–26 change in Diabetes Treatment Satisfaction Questionnaire (DTSQ)10 total treatment satisfaction score from baseline to week 26 (appendix p 5) Safety Endpoints: Change in bodyweight from baseline to week 26 Mean weekly total insulin dose during weeks 24–26 and 50–52 Percentages of time below 3·0 mmol/L (< 54 mg/dL) and above range (TAR; > 10·0 mmol/L [> 180 mg/dL]) during weeks 22–26 Number of overall clinically significant hypoglycemic episodes (< 3·0 mmol/L [< 54 mg/dL] confirmed by blood glucose meter) Number of overall severe hypoglycemic episodes (associated with severe cognitive impairment requiring external assistance for recovery	Mean HbA1c at week 26: Icodec: 7·59% (0·96%) to 7·15% Degludec: 7·63% (0·93%) to 7·10% Mean change in HbA1c from baseline to week 26: Icodec: − 0·47% Degludec: − 0·51% with an estimated treatment difference (ETD) of 0·05 percentage points (95% CI − 0·13 to 0·23), confirming non-inferiority (*p* = 0·0065) of icodec to degludec Mean HbA1c reduction at week 52: Icodec, − 0·37 percentage points Degludec, − 0·54 percentage points; ETD 0·17 percentage points [95% CI 0·02 to 0·31]; *p* = 0·021) Mean FPG concentrations at week 26: Icodec: − 0·84 mmol/L (− 15 mg/dL) Degludec: − 1·87 mmol/L (− 34 mg/dL) (ETD 1·03 mmol/L [95% CI 0·48 to 1·59], 19 mg/dL [95% CI 9 to 29], *p* = 0·0003) Mean change in FPG at week 52: Icodec: − 0·58 mmol/L (− 10 mg/dL) Degludec: − 1·88 mmol/L (− 34 mg/dL) (ETD 1·30 mmol/L [95% CI 0·73 to 1·86], 23 mg/dL [95% CI 13 to 34], *p* < 0·0001) Percentage of TIR at weeks 22–26 Icodec: 59.1% (14 h and 11 min) Degludec: 60.8% (14 h and 36 min) (ETD − 2·00% [95% CI − 4·38 to 0·38], *p* = 0·099) Change in Diabetes Treatment Satisfaction Questionnaire (DTSQ)10 at week 26 Icodec: 1.97 Degludec: 3.06 ETD − 1·09 [95% CI − 1·85 to − 0·34], *p* = 0·0044) Change in bodyweight from baseline to week 26 Icodec: 1.29 kg Degludec: 1.01 kg (ETD 0·28 kg [95% CI − 0·37 to 0·92], *p* = 0·41) Mean weekly total insulin dose during weeks 24–26 and 50–52 Weeks 24–26: Icodec 311 U/week [∼44 U/day] Degludec, 323 U/week [∼46 U/day] ETR: 0·96 [95% CI 0·90 to 1·03], *p* = 0·27) Weeks 50–52 Icodec: 310 U/week [∼44 U/day] Degludec, 329 U/week [∼47 U/day]; ETR 0·94 [95% CI 0·88 to 1·01], *p* = 0·11) Percentages of time below 3·0 mmol/L (< 54 mg/dL) and above range (TAR; > 10·0 mmol/L [> 180 mg/dL]) during weeks 22–26 Icodec, 1.0% (15 min/day) Degludec, 0.7% (10 min per day]; [ETR], 1.46 [95% CI 1·16—1·85], *p* = 0·0014) Rates of combined clinically significant or severe hypoglycaemia: week 26: Icodec (19·93 events per patient-year of exposure [PYE]) Degludec (10·37 events per PYE; estimated rate ratio [ERR], 1·89 [95% CI 1·54 to 2·33], *p* < 0·0001) Week 57 weeks: Icodec (17.00 events per PYE) Degludec (9.16 events per PYE; ERR, 1·80 [95% CI 1·48 to 2·18]; *p* < 0·0001)
Arms: once-weekly icodec or once-daily degludec, both in combination with mealtime aspart (two or more daily injections)	randomized, multicenter (99 sites across 12 countries), open-label, active-controlled, parallel-group, treat-to-target, phase 3a		
Population: T1DM treated with multiple daily insulin injections (basal-bolus insulin analogue regimens) for at least 1 year with (HbA1c) < 10·0%			
Published: 2023	Duration: 52 weeks (2-week screening period, a 52-week treatment phase (26-week main phase and 26-week safety extension phase), and a 5-week follow-up period)		
Rosenstock et al. [22]	*N* = 247	Primary Endpoint: absolute change in the glycated hemoglobin level baseline to week 52 Secondary end point: percentage of time spent in the target glycemic range of 70—180 mg per deciliter (3.9 to 10.0 mmol per liter) in weeks 48 to 52, as measured by blinded CGM Secondary efficacy end point: Mean change in the fasting plasma glucose level from baseline to week 52	A1c at Week 52: Icodec: − 1.55% Glargine: − 1.35% estimated treatment difference of − 0.19 percentage points (95% confidence interval (CI − 0.36 to − 0.03) Percentage of time in the target glycemic range at weeks 48–52: Icodec: 71.9% Glargine: 66.9%; estimated treatment difference, 4.27 percentage points [95% (CI 1.92 to 6.62]; *P* < 0.001), (approximately 1 h and 1 min additional time spent in range per day in Icodec vs glargine) Estimated mean change in the fasting plasma glucose level at week 52: Icodec: − 60.32 mg per deciliter [− 3.35 mmol per liter] Glargine: − 60.08 mg per deciliter [− 3.33 mmol per liter]; estimated treatment difference, − 0.24 mg per deciliter [95% (CI, − 4.89 to 4.41), or − 0.01 mmol per liter [95% CI, − 0.27 to 0.24] Percentage of patients reaching a A1c < 7% at week 26: Icodec: 72% Glargine: 68% (estimated odds ratio, 1.20; 95% CI, 0.98 to 2.13) A1c of < 6.5%: Icodec: 49% Glargine: 39%, respectively (estimated odds ratio, 1.47; 95% CI, 0.85 to 2.52)
Arms: Patients were randomly assigned in a 1:1 ratio to receive either once-weekly subcutaneous icodec plus once-daily placebo or once-daily subcutaneous glargine plus once-weekly placebo	randomized, double-blind, double-dummy, treat-to-target, active-controlled, parallel-group, multinational phase 2 trial		
Population: Adults (≥ 18 years of age) with T2DM who had not previously received insulin and who had a glycated hemoglobin level of 7 to 11% (53.0 to 96.7 mmol per mole) and a body-mass index (the weight in kilograms divided by the square of the height in meters) of 40 or less			
Published: 2023	Duration: approximately 85 weeks, comprising a screening period (up to 2 weeks), a 78-week randomized treatment period		
Pieber et al. [23]	*N* = 85	Primary Endpoint: Frequency, timing, and recovery of hypoglycemia after administration of double and triple doses of icodec vs glargine	Clinically significant hypoglycemia (BG < 54 mg/dL): Double glargine dose: 15 (35.7%) Double icodec dose: 17 (39.5%) Triple glargine dose: 28 (70.0%) Triple icodec dose: 20 (52.6%) Severe hypoglycemia (BGnadir < 45 mg/dL): Double glargine dose: 3 (7.1%) Double icodec dose: 2 (4.7%) Triple glargine dose: 10 (25%) Triple icodec dose: 1 (2.6%) (*p* < 0.05) Mean BGnadir: Double glargine dose: 60 mg/dL Double icodec dose: 58 mg/dL Triple glargine dose: 52 mg/dL Triple icodec dose: 56 mg/dL (*p* < 0.05) Time to clinically significant hypoglycemia (BG < 54 mg): Double glargine dose: 4.5 h Double icodec dose: 2.9 h Triple glargine dose: 2.2 h Triple icodec dose: 2.4 h Time to recovery following glucose infusion in patients with severe hypoglycemia (BGnadir to BG): Double glargine dose: 21.0 min Double icodec dose: 28.3 min (*p* < 0.05) Triple glargine dose: 23.3 min Triple icodec dose: 23.8 min Amount of glucose needed in patients with severe hypoglycemia (BGnadir to BG): Double glargine dose: 111 mg/kg Double icodec dose: 141 mg/kg (*p* < 0.05) Triple glargine dose: 115 mg/kg Triple icodec dose: 116 mg/kg
Arms: Icodec vs glargine U-100	Randomized, single study, open-label, two-phase crossover trial		
Population: T2DM			
Published: 2023	Duration: Icodec: 6 weeks Glargine: 11 days		

*DTSQ = Diabetes Treatment Satisfaction Questionairre

**TRIM-D = Treatment Related Impact Measure for Diabetes

Nishimura et al. [11] evaluated pharmacological and molecular properties of insulin icodec. Their *in vitro* cell-based studies revealed the when a C20 fatty diacid-containing side chain is added to insulin icodec, it results in strong, reversible binding to albumin. Additionally, three amino acid substitutions (A14E, B16H and B25H) enhance its molecular stability and reduce insulin receptor binding and clearance, thereby extending the half-life. In a clinical pharmacology trial involving subjects with T2DM, insulin icodec was tolerated well and exhibited pharmacokinetic and pharmacodynamic characteristics suitable for once-weekly dosing. The mean half-life was 196 h, and the glucose-lowering effect was distributed evenly over the entire week-long dosing interval. A limitation was the small number of subjects studied (*n* = 12), however this study provided much value to help confirm the pharmacology and kinetics on insulin icodec.

Pieber et al. [13] compared the timing of the pharmacokinetic profiles of the administration of insulin in an open label study of 46 patients with T2DM. The study showed, in order to reach steady state, 3–4 doses of insulin icodec must be administered. The glucose lowering effects of insulin icodec follows its pharmacokinetic effects in that the most glucose lowering occurs between days 2 and 3 while it slowly tapers down towards the end of the week. However, the overall difference between days is fairly low. This small, open-label study shows insulin icodec can safely be used once a week without great alterations in blood glucose levels.

Plum-Mörschel et al. [10] conducted a randomized, open-label, crossover trial comparing the sites of insulin icodec administration and their pharmacokinetic and pharmacodynamic response. Administration of insulin into the subcutaneous tissue of the abdomen, upper arms, and thigh had similar effects on glucose lowering despite higher peaks when administered in the upper arm or abdomen in comparison to the thigh. Strengths include randomization and both pharmacokinetic (insulin levels) and pharmacodynamic (glucose lowering effect) endpoints. Limitations of the study are its small (*n* = 25 and open label design.

Bajaj et al. [14] conducted a trial to investigate two methods of switching to insulin icodec from once-daily insulin glargine U-100 in patients with T2DM who were using daily basal insulin and one or more oral glucose-lowering medications. This phase 2, randomized (1:1:1) trial involved basal insulin–treated subjects with T2DM (A1C 7.0–10.0%) switching to icodec with an initial 100% loading dose (where only the first dose was doubled), switching to icodec without a loading dose, or continuing insulin glargine U-100 for 16 weeks. The results showed that switching from daily basal insulin to once-weekly icodec did not exhibit many adverse effects provided effective glucose control in both insulin icodec groups. The rates and incidences of adverse effects and hypoglycemic episodes were similar across the groups. A loading dose at initiation when switching to once-weekly icodec significantly increased the percentage of TIR during weeks 15 and 16 compared to once-daily IGlar U100, without increasing the risk of hypoglycemia. The trial’s limitations included its modest sample size and relatively short duration. However, it had many strengths such as it multicenter and randomized design, low treatment discontinuation rate, and use of CGM.

Lingvay et al. [15] studied the safety and efficacy of insulin icodec using various once-weekly titration algorithms in a phase 2, open-label, randomized, 16-week, treat-to-target study. The trial included adults that were insulin-naive (*n* = 205) with T2DM and A1C levels of 7–10% who were on oral glucose-lowering medications. Participants began once-weekly icodec and were titrated weekly using different algorithms which varied in FBG goal ranges and dosing increments. The percentage of TIR during weeks 15 and 16 was monitored using CGM continuous glucose monitoring. The study found that once-weekly icodec was well tolerated and effective across the three titration algorithms. The titration algorithm A (FBG goal: 80–130 mg/dL; ± 21 units/week) achieved the greatest balance between glycemic control and hypoglycemia risk. However, titration B (± 28 units/week with the same FBG goal) improved TIR but resulted in more hypoglycemia. The strengths of this study were the multicenter and randomized design, low treatment discontinuation amount, and assessment of glycemic control using TIR with CGM data. ​​The main limitation of this study was its short length of 16 weeks.

Philis-Tsimikas et al. [12] authored a design paper describing the characteristics and rationales of the ONWARDS series. The primary glycemic outcomes measured were the change in A1C from the beginning of the study to week 26, A1C differences from baseline to week 52, change in FBG, and TIR. Additionally, all studies compared safety outcomes, namely hypoglycemia. Insulin icodec was studied in various populations including T2DM (ONWARDS 1–5) [16–20], insulin-naive patients (ONWARDS 1, 3, 5) [16, 18, 20], previously insulin-treated patients (ONWARDS 2, 4) [17, 19], and T1DM (ONWARD 6) [21]. Finally, the series compared insulin icodec to various other insulins including insulin glargine U-100 (ONWARDS 1,4, 5) [16, 19, 20], concentrated insulin glargine U-300 (ONWARDS 5) [20], and long acting insulin degludec (ONWARDS 2, 3, 5, 6) [17, 18, 20, 21]. Overall, this composition of studies allows for broad generalizability for patients with both T1DM and T2DM who either had been on insulin or were insulin naive compared to multiple different basal insulins.

Rosenstock et al. [16] evaluated once-weekly insulin icodec in comparison to once-daily insulin glargine U100 in patients with T2DM that were not previously taking insulin (ONWARDS 1). A1C was reduced in patients taking insulin icodec compared to patients taking insulin glargine and both noninferiority and superiority of insulin icodec were confirmed. Insulin icodec also showed a higher TIR compared to insulin glargine. Hypoglycemic events were not statistically different at 52 weeks but icodec was inferior to glargine at 83 weeks. This is the longest trial studying insulin icodec so far. Strengths were the extended duration of the study, randomized treatment methods which included safety follow-up. Limitations were that this trial did not include a double-blind, double-dummy design and the authors stated the reason was because of the intent to limit the confounding effects on trial subjects from the number of injections that would be needed over the course of the extended duration of the study.

Another Philis-Tsimikas et al. [17] study evaluated insulin icodec to daily insulin degludec in type 2 patients already taking basal insulin (ONWARDS 2). Overall the effect on A1C from baseline to week 26 was greater with insulin icodec than insulin degludec which showed not only a non-inferiority but also superiority of icodec compared to degludec. Weight gain was significantly higher in subjects taking icodec versus degludec and the combined level 2 and 3 hypoglycemia were similar. Compared with degludec, subjects receiving icodec had higher percentages of A1C less than 7% without clinically significant hypoglycemia, and a greater improvement in Diabetes Treatment Satisfaction Questionnaire (DTSQ). The strengths of the study included its randomized, large, multi-center design with diverse participants, high trial completion percentage, use of double-blinded CGM, and gathering of patient feedback on diabetes treatment satisfaction. However, several limitations should be noted. The open-label design could introduce bias into patient-reported parameters and the report of hypoglycemia. Additionally, the trial was powered to evaluate the primary outcome, so any statistically significant differences between groups for secondary outcomes might not be from true clinical effect.

Another study by Lingvay et al. [18] evaluated once-weekly insulin icodec versus once-daily insulin degludec in subjects with T2DM that were not previously taking insulin (ONWARDS 3). A1C was significantly reduced in patients taking insulin icodec compared to patients taking insulin degludec and both noninferiority and superiority of insulin icodec were confirmed. Episodes of hypoglycemia were found to be greater with subjects taking insulin icodec compared to degludec but these values were not statistically significant. There was no discernable difference in weight between the two study groups. The study’s strengths included its double-masked, double-dummy design, and high trial completion rate, which ensured it was well-powered to assess the primary endpoint. However, the study had several limitations. Its 26 weeks duration meant that sustained effects could not be predicted. Additionally, to allow a bases of comparison between the two groups, both insulins were titrated on a weekly basis, whereas conventional adjustment of once-daily degludec could occur daily. Another limitation of the trial was that it was only powered to evaluate the primary outcome, so any statistical differences between the two groups for secondary outcomes, including hypoglycemia, does not necessarily reflect a clinical effect. Lastly, this trial did not use CGM to study TIR as a parameter.

Mathieu et al. [19] evaluated a group of type 2 subjects already taking basal-bolus insulin to assess any differences in being on basal insulin glargine versus basal insulin icodec (ONWARDS 4). This study evaluated adults from 80 sites across nine countries. Overall, both insulin glargine and insulin icodec reduced A1C from baseline to week 26 but was not statistically significantly different and showed a non-inferiority of icodec compared to glargine. All adverse effects were lumped together so it was difficult to assess differences but numerically they appeared to be similar. Also similar were the reported rates of level 2 and level 3 hypoglycemia. The strengths of this study included blinded CGM, high trial completion rate, and inclusion of large, multinational, diverse subjects. However, the trial had some limitations. It was short in duration and had an open-label design. The authors did note that the open-label design was selected for safety reasons, as it was not possible to blind two treatment groups without risking confusion between the two basal pens and the one bolus pen in a double-blind, double-dummy design.

Bajaj et al. [20] also compared a group of insulin-naive subjects with T2DM to assess differences between taking insulin icodec with a predetermined dosing guide app and using daily basal insulin analogues (degludec, glargine U100, glargine U300, selected by study investigator at initial screening) (ONWARDS 5). This study involved adults from seven countries and 176 sites. The results showed that the effect on A1C from baseline to week 52 was higher with insulin icodec using the app than daily basal insulin analogues. This difference was significant, demonstrating not only non-inferiority but also superiority of insulin insulin icodec with app use. Additionally, the DTSQ total treatment satisfaction score for week 52 was significantly higher with icodec and the app compared to daily basal analogues. The Treatment Related Impact Measure for Diabetes (TRIM-D) domain score that measures compliance was also statistically greater for icodec with app. Strengths of this study were its broad inclusion criteria, investigator discretion on choice and dose of daily basal analogues, and individualized trial site visit schedules which reflect clinical practice in the real-world. Limitations were plenty. It had an open-label trial design which could have brought in bias on how the trial was conducted. The authors also note that although it was a 52 week trial, it was not long enough to evaluate diabetes-related and cardiovascular disease outcomes related to the use of insulin icodec.

Russel-Jones et al. [21] evaluated the use of once-weekly insulin icodec compared to once-daily insulin degludec in T1DM participants as part of a basal-bolus set up in a 52 week randomized, open label phase 3a trial across 12 countries (ONWARD6). Adults with T1DM with A1C < 10.0% were randomized to (1:1) to once-weekly icodec or once-daily degludec, both which were in combination with insulin aspart (two or more daily injections). Primary endpoints were change in A1C from baseline to week 26 and test for non-inferiority. The results of the study showed non-inferiority of icodec to degludec. Strengths of the study were its multicentered design, inclusion of patients with T1DM, use of CGM monitors, and long duration. Limitations of the study included open-label design which could lead to risk of potential bias and the utilization of self-measured blood glucose readings compared to CGM for insulin dose adjustments.

Rosenstock et al. [22] also conducted a 78-week randomized, open-label, treat-to-target phase 3a trial in adults with T2DM (A1C 7- 11%) who had not previously been given insulin. Participants were randomized to a 1:1 ratio to receive once-weekly insulin icodec or once-daily insulin glargine U-100. The primary endpoint were the differences in A1C from baseline to week 52. The total mean reduction in A1C at 52 weeks was higher with icodec compared to with insulin glargine, which confirms the noninferiority and superiority of icodec. Overall the rates of hypoglycemic episodes appeared to be less than one event per person-year of exposure for the entire trial. This trial did have blinding of glucose measurements and a long duration, however it had a low proportion of Black and Latino participants.

Pieber et al. [23] also conducted a randomized, open-label, cross-sectional study comparing hypoglycemia in insulin icodec and insulin glargine treated subjects. The study involved a run in period to calculate a dose for the patients. The patients were then administered both double and triple their normal dose for icodec and for glargine. Next, patients were monitored to compare hypoglycemia trends. Once hypoglycemic, the patients were administered intravenous glucose to achieve euglycemia. The proportion of patients achieving clinically significant hypoglycemia (BG < 54 mg/dL) in the two groups were similar. However, patients receiving triple dosing of glargine achieved higher levels of severe hypoglycemia (BG < 45 mg/dL) compared to those receiving triple doses of icodec. Additionally, those taking triple doses of glargine had lower nadir blood glucose compared to icodec. Alternatively, those taking double doses of icodec took longer and needed more glucose to recover from hypoglycemia compared to insulin glargine. Time to achieve clinically significant hypoglycemia was similar between the two groups. Counter-regulatory hormones (namely adrenaline and cortisol) were greater in the triple dose of icodec vs glargine. Finally, symptoms (mental status and vitals) changed in a similar fashion between the two groups. Although small and open-label, this study concluded that similar hypoglycemic reactions occur between icodec and glargine.

## Effectiveness

Overall in Type 2 diabetes patients participating in the ONWARDS 1, 2, 3, and 5 trial, the effect of insulin icodec showed a significantly greater A1C reduction from baseline compared to insulin degludec, glargine U100, and glargine U300. Moreover, these analyses affirmed both the noninferiority and superiority of insulin icodec relative to the referenced comparators [16–18, 20]. In the ONWARDS 6 trial, which involved individuals with Type 1 diabetes, insulin icodec was found to be noninferior to insulin degludec in terms of A1C reductions during the main phase of the study, from baseline to week [21]. At the end of the extension phase (week 52), a statistically significant greater mean reduction in A1C was observed with insulin icodec compared to degludec.

## Warnings, precautions, contraindications

The warnings, precautions, and contraindications for insulin icodec mirror any basal insulin product. Risk of hypoglycemia is increased with concurrent use of other insulin agents as well as insulin secretagogues. Risk of hypoglycemia is enhanced from lack of food or excess exercise. Injection site irritation is also noted. If patients are allergic or hypersensitive to insulin icodec or any of its components it should not be used. Patients with renal or liver dysfunction should check their glucose more frequently when using insulin icodec.

## Adverse effects

When considering side effects of insulin, the concerns which are most commonly reported are injection site reaction, hypoglycemia and weight gain. In the ONWARDS trials, hypoglycemic events were categorized as alert level 1 (≥ 54 to < 70 mg/dL), clinically significant level 2 (< 54 mg/dL), or severe level 3 (associated with significant cognitive impairment requiring external help for recovery), with nocturnal hypoglycemic events defined as those occurring between 00:01 am and 05:59 am. Importantly, similar rates of combined level 2 or level 3 hypoglycemia were observed in patients with Type 2 diabetes across ONWARDS 1 through 5 [16–20]. There was no observed increase in the rate of combined level 2 or 3 hypoglycemia between insulin icodec and daily basal insulin comparators from baseline to week 26 in the ONWARDS 1, 2, 4, and 5 trials [16, 17, 19, 20]. However, in the ONWARDS 3 study [18], a statistically significant difference in hypoglycemia was noted among those treated with icodec at week 26, but this difference was not observed at the end of the study at week 31. Overall some studies did find that insulin icodec had a greater number of clinically significant hypoglycemic events, while other studies did not show significant differences. In ONWARDS 6 [21] and other similar studies, higher rates of clinically significant or severe hypoglycemic episodes were observed in the icodec group than the degludec group during the on-treatment period [21, 22]. In other studies, such as a study by Nishimura E et al. [11], insulin icodec was well tolerated with no increase in adverse event incidence with increasing insulin icodec doses. Prolonged hypoglycemia is also a concern. A study by Pieber et al. [23] examined time to hypoglycemia and time to recovery with glucose infusion. Results from the trial showed similar duration of hypoglycemic episodes with icodec vs glargine U100 in type 2 diabetes and recovery from hypoglycemia by constant intravenous glucose infusion took < 30 min for all treatments. Injection site reactions were also noted [22].

In terms of weight gain, similar to hypoglycemia, study results are mixed. One study by Bajaj et al. [14] showed a greater increase in body weight with icodec without loading dose compared with IGlar U100 (ETD, 1.22 kg [95% CI 0.24–2.2]; *P* = 0.01). Another study by Philis-Tsimikas et al. [17] showed icodec caused weight gain of + 1.40 kg while degludec caused weight loss of −0.30 kg (95% CI, −0.76 to 2.63). However, a few studies including a study by Lingvay et al. [17] did not observe a difference in weight change between icodec and degludec at week 26. Based on the mixed data, it is uncertain if hypoglycemia and weight gain occur at similar rates in comparison to other insulins.

## Drug interactions

As an insulin analog, insulin icodec does not have many drug interactions given the medication is a peptide that is not renally adjusted or metabolized by the liver and remembering that insulin is endogenously created by the body. Like all insulins, however, certain pharmacodynamic parameters should be considered. First, insulins may have synergistic effects when used with other diabetes agents. Although most commonly seen with short acting insulins and insulin secretagogues, using concurrent medications that sensitize the body to insulin, such as thiazolidinediones, can increase risk of hypoglycemia. Similarly, any medications that have an adverse effect of hyper or hypoglycemia may have subtractive or additive glycemic effects. For example, a patient stable on insulin icodec may seem to be no longer controlled with the medication if they are started on a corticosteroid that can increase blood glucose. Even lifestyle factors such as exercise, stress, or alcohol consumption can cause similar effects and should be monitored. Additionally, patients themselves have different sensitivities to insulin especially patients with T2DM. Finally, clinicians using insulin analogs or any diabetes medication for their patients should recall that beta-blockers can mask symptoms of hypoglycemia.

Theoretical interactions include medications and disease states that alter albumin levels since insulin icodec utilizes albumin to lengthen its duration of action. Though not yet seen in clinical practice, management of these conditions and any of the aforementioned interactions is done simply by monitoring and adjusting insulin doses based on changes in blood glucose levels. Some clinicians may also adjust insulin levels to prevent hypoglycemia when adding on medications such as thiazolidinediones or glucagon-like peptide-1 receptor agonists (GLP-1 RAs).

## Dosage and administration

Insulin icodec use results in less injections by 86%, 52 injections instead of 365 injections annually. Once weekly insulin icodec also fits into the administration frequency of once-weekly incretin analogues, and this combination would be effective from a weight, hypoglycemia, and cardiovascular benefit perspective [1].

**Fig. 2:**
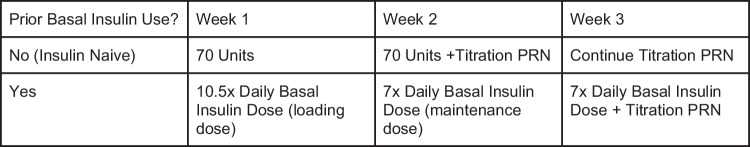
Insulin Icodec Initial Dosing for Type 2 [12, 24]

**Fig. 3:**
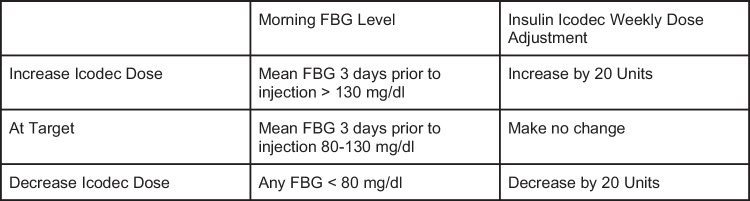
Insulin Icodec Titration [12, 24]

The concentration of insulin icodec is available as 700 units per milliliter (U700) in a pre-filled pen injector. This allows for the same injection volume of 100 units per milliliter (U100). It takes 3–4 weeks for insulin icodec to reach steady state and as a result a one time loading dose is recommended to reach steady state quicker, although we will await the drug manufacturer package insert for specifics. For insulin naive patients with T2DM, the ONWARDS trials suggest a starting dose of 70 units once-weekly, which is equivalent to 10 units of daily basal insulin. If upward or downward titration is needed, the recommendation is 20 units weekly if fasting blood glucose is above or below the target range 3 days prior to the next injection. Because of the one time loading dose, titration can occur weekly rather than waiting for 3–4 weeks to reach steady state. Although titrating basal insulin by 20 unit increments seems aggressive, this titration for insulin icodec would be similar to 3 unit adjustments daily using traditional long acting basal insulin. If a switch from traditional basal insulin to insulin icodec is needed, the initial dose is 7 times the previous daily dose of basal insulin after a one-time loading dose of additional 50% of the calculated once-weekly dose which is the same as 10.5 times the daily basal dose as a loading dose to get to steady state more rapidly. At week 2, the recommended dose is 7X the previous daily dose, with on-going weekly titration by 20 unit increments starting week 3. If a risk of hypoglycemia is identified, a 10 unit per week titration would be less aggressive and reduce risk [12, 24]. See Figs. 2 and 3.

**Fig. 4:**
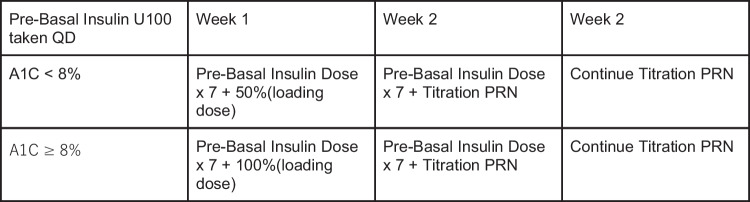
Insulin Icodec Initial Dosing for Type 1 taking Pre-Basal Insulin U100 Taken Daily [21]

**Fig. 5:**
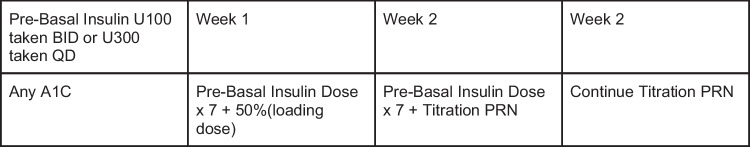
Insulin Icodec Initial Dosing for Type 1 taking Pre-Basal Insulin U100 Taken Twice Daily or U300 Taken Once Daily [21]

Dosing for type 1 is based on ONWARDS 6 [21]. Since all patients included in the trial were already on basal insulin there were no recommendations for the starting dose for insulin naive patients. For the first injection after switching from another basal insulin, icodec dose was based on pre basal dose multiplied by 7 plus a one-time additional 50% loading dose if A1C was less than 8% or a one-time additional 100% loading dose if A1C was greater than or equal to 8%. See Fig. 4. Subjects previously using insulin glargine U300 or twice daily basal insulin were given a one-time additional 50% loading dose regardless of their A1C. See Fig. 5. For the second injection and thereafter subjects were given the pre basal dose multiplied by 7 plus titration. See Fig. 3.

Similar to other insulins, icodec is administered subcutaneously. The administration of insulin into the subcutaneous tissue of the abdomen, upper arms, and thigh had similar effects on glucose lowering despite higher peaks when administered in the upper arm or abdomen compared to the thigh [10]. Missed doses will be addressed by the FDA labeling and manufacturer which has yet to be finalized.

## Regulatory issues

Insulin icodec is to be administered subcutaneously at the abdomen, thigh or upper/outer arm (back) or buttock. Insulin icodec should be stored similar with other types of insulins. The insulin should be stored in the refrigerator within a range of 2 °C and 8 °C for long-term storage until the expiration date as recommended by FDA. There has not been official information released on the shelf life on icodec after it has been opened though current basal insulins range from 28–56 days [25]. Insulin icodec is formulated as 700 units/mL to ensure that the injection volume is similar to that of once-daily basal insulin. It is designed to be injected subcutaneously once a week with an easy-to-use pen. Patients can also use a digital support solution for personalized automated guidance [26]. Since this medication is not yet FDA approved, pricing and coverage is not yet determined.

## Conclusion

With the approval of insulin icodec, health care providers and patients with T1DM or T2DM needing insulin will have a new tool to help manage their blood glucose. The novel formulation allows for a prolonged duration of activity which leads to once weekly dosing of insulin that should improve compliance and satisfaction in patients needing to inject insulin. The initiation and titration of this novel formulation differs from traditional insulins and requires more initial attention from both providers and patients but the benefits of once weekly dosing will improve quality of life. The reviewed literature showed similar and sometimes improved glycemic control when insulin icodec was compared to other long-acting insulins both in insulin-naive and previously insulin-treated patients with the data being most robust in patients with type 2 diabetes. Although icodec was studied in type 1 diabetes we will need more data from future trials to observe its full advantage. It is unknown whether the improvement in glycemic control were due to differences in the insulins or due to improved compliance with once weekly dosing since the studies used an intention-to-treat analysis. Finally, hypoglycemia was similar or slightly increased with insulin icodec when compared to other long acting insulins, with again, most studies conducted in type 2 patients and much less studies conducted in type 1 diabetes. Overall, icodec is a useful, new formulation of basal insulin that allows for less injections, improved compliance, and potentially improved glycemic control providing a new tool to practitioners managing patients with diabetes who need to be on insulin.

## Data Availability

No datasets were generated or analysed during the current study.

## References

[1] Singh AK, Singh A, Singh R, et al. Once-weekly basal insulin icodec: Looking ONWARDS from pharmacology to clinical trials. Diabetes Metab Syndr. 2022;16:102615. 10.1016/j.dsx.2022.102615. 36108418 10.1016/j.dsx.2022.102615

[2] Polonsky WH, Fisher L, Hessler D, et al. Patient perspectives on once-weekly medications for diabetes. Diabetes Obes Metabol. 2011;13:144–9. 10.1111/j.1463-1326.2010.01327.x 21199266

[3] Abuelazm M, Ibrahim A, Khlidj Y, et al. Once-weekly insulin icodec versus once-daily long-acting insulin for type 2 diabetes: A meta-analysis of randomized controlled trials. J Endocr Soc. 2024;8:1–13. 10.1210/jendso/bvad177 PMC10783254 38213906

[4] Sugumar V, Ang KP, Alshanon AF, et al. A comprehensive review of the evolution of insulin development and its delivery method. Pharmaceutics. 2022;14:1406. 10.3390/pharmaceutics14071406. 35890301 10.3390/pharmaceutics14071406 PMC9320488

[5] Arnolds S, Kuglin B, Kapitza C, et al. How pharmacokinetic and pharmacodynamic principles pave the way for optimal basal insulin therapy in type 2 diabetes. Int J Clin Pract. 2010;64:1415–24. 10.1111/j.1742-1241.2010.02470. 20618882 10.1111/j.1742-1241.2010.02470.x PMC2984539

[6] Lucidi P, Porcellati F, Andreoli AM, et al. Pharmacokinetics and pharmacodynamics of NPH insulin in type 1 diabetes: The importance of appropriate resuspension before subcutaneous injection. Diabetes Care. 2015;38:2204–10. 10.2337/dc15-0801. 26358287 10.2337/dc15-0801

[7] Furman BL, Glargine Insulin, Reference Module in Biomedical Sciences, Elsevier, 2017, ISBN 9780128012383, 10.1016/B978-0-12-801238-3.97985-6

[8] Chapman TM, Perry CM. Insulin detemir: a review of its use in the management of type 1 and 2 diabetes mellitus. Drugs. 2004;64:2577–3259. 10.2165/00003495-200464220-00008. 15516157 10.2165/00003495-200464220-00008

[9] Nasrallah SN, Reynolds LR. Insulin degludec, the new generation basal insulin or just another basal insulin? Clin Med Insights Endocrinol Diabetes. 2012;5:31–7. 10.4137/CMED.S9494. 22879797 10.4137/CMED.S9494 PMC3411522

[10] Plum-Mörschel L, Andersen LR, Hansen S, et al. Pharmacokinetic and pharmacodynamic characteristics of insulin Icodec after subcutaneous administration in the thigh, abdomen or upper arm in individuals with type 2 diabetes mellitus. Clin Drug Investig. 2023;43:119–27. 10.1007/s40261-022-01243-6. 10.1007/s40261-022-01243-6 PMC9902323 36631720

[11] Nishimura E, Pridal L, Glendorf T, et al. Molecular and pharmacological characterization of insulin icodec: a new basal insulin analog designed for once-weekly dosing. BMJ Open Diabetes Res Care. 2021;9:e002301. 10.1136/bmjdrc-2021-002301. 10.1136/bmjdrc-2021-002301 PMC8378355 34413118

[12] Philis-Tsimikas A, Bajaj HS, Begtrup K, et al. Rationale and design of the phase 3a development programme (ONWARDS 1–6 trials) investigating once-weekly insulin icodec in diabetes. Diabetes Obes Metab. 2023;25:331–41. 10.1111/dom.14871. 36106652 10.1111/dom.14871 PMC10092674

[13] Pieber TR, Asong M, Fluhr G, et al. Pharmacokinetic and pharmacodynamic properties of once-weekly insulin icodec in individuals with type 2 diabetes. Diabetes Obes Metab. 2023;25:3716–23. 10.1111/dom.15266. 37694740 10.1111/dom.15266

[14] Bajaj HS, Bergenstal RM, Christoffersen A, et al. Switching to once-weekly insulin Icodec versus once-daily insulin glargine U100 in type 2 diabetes inadequately controlled on daily basal insulin: A phase 2 randomized controlled trial. Diabetes Care. 2021;44:1586–94. 10.2337/dc20-2877. 33875485 10.2337/dc20-2877 PMC8323191

[15] Lingvay I, Buse JB, Franek E, et al. A Randomized, open-label comparison of once-weekly insulin Icodec titration strategies versus once-daily insulin glargine U100. Diabetes Care. 2021;44:1595–603. 10.2337/dc20-2878. 33875484 10.2337/dc20-2878 PMC8323172

[16] Rosenstock J, Bain SC, Gowda A, et al. Weekly Icodec versus daily glargine U100 in type 2 diabetes without previous insulin. N Engl J Med. 2023;389:297–308. 10.1056/NEJMoa2303208.2023. 37356066 10.1056/NEJMoa2303208

[17] Philis-Tsimikas A, Asong M, Franek E, et al. Switching to once-weekly insulin icodec versus once-daily insulin degludec in individuals with basal insulin-treated type 2 diabetes (ONWARDS 2): a phase 3a, randomised, open label, multicentre, treat-to-target trial. Lancet Diabetes Endocrinol. 2023;11:414–25. 10.1016/S2213-8587(23)00093-1. 37148899 10.1016/S2213-8587(23)00093-1

[18] Lingvay I, Asong M, Desouza C, et al. Once-weekly insulin Icodec vs once-daily insulin degludec in adults with insulin-naive type 2 diabetes: The ONWARDS 3 randomized clinical trial. JAMA. 2023;330:228–37. 10.1001/jama.2023.11313. 37354562 10.1001/jama.2023.11313 PMC10354685

[19] Mathieu C, Ásbjörnsdóttir B, Bajaj HS, et al. Switching to once-weekly insulin icodec versus once-daily insulin glargine U100 in individuals with basal-bolus insulin-treated type 2 diabetes (ONWARDS 4): a phase 3a, randomised, open-label, multicentre, treat-to-target, non-inferiority trial. Lancet. 2023;401:1929–40. 10.1016/S0140-6736(23)00520-2. 37156252 10.1016/S0140-6736(23)00520-2

[20] Bajaj HS, Aberle J, Davies M, et al. Once-weekly insulin Icodec with dosing guide app versus once-daily basal insulin analogues in insulin-naive type 2 diabetes (ONWARDS 5): A randomized trial. Ann Intern Med. 2023;176:1476–85. 10.7326/M23-1288. 37748181 10.7326/M23-1288

[21] Russell-Jones D, Babazono T, Cailleteau R, et al. Once-weekly insulin icodec versus once-daily insulin degludec as part of a basal-bolus regimen in individuals with type 1 diabetes (ONWARDS 6): a phase 3a, randomised, open-label, treat-to-target trial. Lancet. 2023;402:1636–47. 10.1016/S0140-6736(23)02179-7. 37863084 10.1016/S0140-6736(23)02179-7

[22] Rosenstock J, Bajaj HS, Janež A, et al. Once-weekly insulin for type 2 diabetes without previous insulin treatment. N Engl J Med. 2020;383:2107–16. 10.1056/NEJMoa2022474. 32960514 10.1056/NEJMoa2022474

[23] Pieber TR, Arfelt KN, Cailleteau R, et al. Hypoglycaemia frequency and physiological response after double or triple doses of once-weekly insulin icodec vs once-daily insulin glargine U100 in type 2 diabetes: a randomised crossover trial. Diabetologia. 2023;66:1413–30. 10.1007/s00125-023-05921-8. 37308751 10.1007/s00125-023-05921-8 PMC10317887

[24] Bajaj HS, Goldenberg RM. Insulin icodec weekly: A basal insulin analogue for type 2 diabetes. TouchREV Endocrinol. 2023;19:4–6. 10.17925/EE.2023.19.1.4 PMC10258611 37313230

[25] https://www.hdrxservices.com/insulin-expiration-dates-an-update/. Last accessed: June 2023

[26] Available at: https://www.novonordisk.com/content/nncorp/global/en/news-and-media/news-and-ir-materials/news-details.html?id=138024. Last accessed: June 2023

